# Preregistered multi-site preclinical randomized controlled trials: the beginning of a new day in reverse translational science?

**DOI:** 10.1038/s41386-025-02090-7

**Published:** 2025-03-20

**Authors:** Lorenzo Leggio, Giancarlo Colombo

**Affiliations:** 1 Clinical Psychoneuroendocrinology and Neuropsychopharmacology Section, Translational Addiction Medicine Branch, National Institute on Drug Abuse Intramural Research Program and National Institute on Alcohol Abuse and Alcoholism Division of Intramural Clinical and Biological Research, National Institutes of Health, Baltimore, MD USA; 2 Neuroscience Institute, Section of Cagliari, National Research Council of Italy, Monserrato, CA Italy

**Keywords:** Drug development, Pharmacology

Despite significant developments in basic neuroscience and pharmacology of alcohol use disorder (AUD), no new medications have been approved for AUD in the past two decades. While there is no single reason for what is often referred to as the ‘valley of death’ in medication development, this challenge requires novel, creative approaches. It is not unusual for a medication tested in animal models of excessive alcohol use to show a positive result, which is then confirmed in initial small human studies. However, multi-site randomized controlled trials (RCTs) often fail to demonstrate medication efficacy. One key question is how to predict early on whether a medication is likely to show efficacy in multi-site RCTs, so that medication development can be faster and more cost-effective. Despite the discovery of several hundred pharmacological compounds that suppress alcohol-related behaviors in laboratory rodents, the number of approved pharmacotherapies can be counted on one hand.

In a recent paper in *Neuropsychopharmacology*, Meinhardt and colleagues [1] propose a new medication development approach based on a pre-registered multi-site preclinical RCT (preRCT). They tested ketamine and R-ketamine using the alcohol deprivation effect model in Wistar rats across three European research centers. The alcohol deprivation effect refers to the temporary increase in alcohol intake after a period of abstinence. The authors showed that ketamine decreased alcohol relapse-like drinking. The authors follow up on the initial findings with post-hoc follow-up experiments in which they compared the ketamine’s results to historical published data testing the FDA-approved acamprosate in the alcohol deprivation effect model.

There are many strengths to the innovative approach of Meinhardt and colleagues [1]. Among these, we would like to highlight two key aspects. The first is the pre-registration. The distinction between postdiction and prediction is critically important in biomedical research, as it is key to improving transparency and credibility of research findings, and to reducing bias and lack of replication [2]. While pre-registration is now common in clinical research, it is still largely absent in preclinical research. This was not always the case in clinical research, but pre-registration is now widely accepted, routinely implemented, and even mandated by several regulatory bodies. Making pre-registration the norm in preclinical research will be crucial to improving transparency, rigor, and reproducibility [2].

Another strength of Meinhardt et al.’s study [1] is their effort to conduct most of the experiments as a coordinated multi-site effort across three sites (Germany, Italy, France). This is an important novelty of their work, leading to additional strengths, such as use of large sample sizes and having multiple investigators and labs adopting the same shared and rigorous experimental designs and procedures. This approach is rarely used in preclinical research but is highly translational, as large multi-site clinical RCTs are often required by regulatory agencies.

As always, the question is: *What next?* Several future directions could be considered in conducting pre-registered multi-site preRCTs. We will briefly discuss four examples below. First, it will be important to standardize alcohol parameters across sites as much as possible, including ensuring consistent and comparable baseline intake of pharmacologically relevant amounts of alcohol. This approach mirrors the use of baseline alcohol drinking levels as inclusion criteria in clinical RCTs. This should be coupled with the use of blood alcohol concentrations and/or other biomarkers of alcohol consumption.

Another aspect in human RCTs is the heterogeneity of patients enrolled in clinical trials. One way to address this challenge in preclinical studies is to use strains of rodents that are not genetically homogeneous. For example, a recent study by Khun and colleagues [3] used heterogeneous stock rats across two sites (USA and Italy) to study extended access heroin self-administration and relapse. They distinguished between vulnerable and resilient rats, and among the vulnerable ones, they further identified subpopulations with distinct opioid-related behaviors [3].

Third, preclinical studies should ideally examine more than one alcohol-related behavior. The alcohol-related behaviors investigated in preclinical pharmacological studies tend to model or mimic single, limited aspects of a complex human disease like AUD. For example, the alcohol deprivation effect model examines relapse to alcohol drinking, as in Meinhardt and colleagues [1], the drinking-in-the-dark binge-like drinking model mimics human binge drinking up to alcohol intoxication [4], and so on. Expanding experimental procedures in multi-site preRCTs would provide more insights into translational potential and better align preRCTs with human RCTs, where multiple alcohol-related behaviors are typically investigated as part of the a priori primary and secondary outcomes.

Lastly, a fourth important challenge, as pointed out by Meinhardt and colleagues [1], is the often-large placebo effect in multi-site clinical RCTs and how to account for this in multi-site preRCTs. The placebo response varies considerably across RCTs and across sites within the same RCT, and it is negatively correlated with treatment effect size [5]. This factor will need to be considered in future preRCTs, although the question remains *how*.

In conclusion, the work by Meinhardt and colleagues [1] is a valuable and important contribution to the addiction literature, offering ideas for novel and exciting future directions. Furthermore, this work serves as a crucial reminder of the importance of expanding clinically inspired reverse translational approaches to develop new, effective treatments for individuals with alcohol and substance use disorders.

## References

[1] Meinhardt MW, Skorodumov I, Jeanblanc J, Benvenuti F, Hilal FF, Domi E, et al. A new module in the drug development process: preclinical multi-center randomized controlled trial of R-ketamine on alcohol relapse. Neuropsychopharmacology. 2025. 10.1038/s41386-025-02071-w. 10.1038/s41386-025-02071-w PMC12032358 40021941

[2] Nosek BA, Ebersole CR, DeHaven AC, Mellor DT. The preregistration revolution. Proc Natl Acad Sci USA. 2018;115:2600–6. 29531091 10.1073/pnas.1708274114 PMC5856500

[3] Kuhn BN, Cannella N, Crow AD, Lunerti V, Gupta A, Walterhouse SJ, et al. Distinct behavioral profiles and neuronal correlates of heroin vulnerability versus resiliency in a multi-symptomatic model of heroin use disorder in rats. Am J Psychiatry. 2025;182:198–208. 39810557 10.1176/appi.ajp.20230623

[4] Rhodes JS, Best K, Belknap JK, Finn DA, Crabbe JC. Evaluation of a simple model of ethanol drinking to intoxication in C57BL/6J mice. Physiol Behav. 2005;84:53–63. 15642607 10.1016/j.physbeh.2004.10.007

[5] Litten RZ, Castle IJ, Falk D, Ryan M, Fertig J, Chen CM, et al. The placebo effect in clinical trials for alcohol dependence: an exploratory analysis of 51 naltrexone and acamprosate studies. Alcohol Clin Exp Res. 2013;37:2128–37. 23889231 10.1111/acer.12197 PMC3823636

